# Cognitive behavioral phenotyping of *DSCAM* heterozygosity as a model for autism spectrum disorder

**DOI:** 10.1111/gbb.70002

**Published:** 2024-09-18

**Authors:** Ryan C. Neff, Katherine A. Stangis, Ujjawal Beniwal, Ty Hergenreder, Bing Ye, Geoffrey G. Murphy

**Affiliations:** ^1^ Michigan Neuroscience Institute, University of Michigan Medical School Ann Arbor Michigan USA; ^2^ Department of Molecular & Integrative Physiology University of Michigan Ann Arbor Michigan USA; ^3^ Life Sciences Institute, University of Michigan Ann Arbor Michigan USA; ^4^ Department of Cell and Developmental Biology University of Michigan Ann Arbor Michigan USA

**Keywords:** autism spectrum disorder, behavior, *DSCAM*, fear learning, learning and memory, spatial learning

## Abstract

It is estimated that 1 in 36 children are affected by autism spectrum disorder (ASD) in the United States, which is nearly a twofold increase from a decade ago. Recent genetic studies have identified de novo loss‐of‐function (dnLoF) mutations in the *Down Syndrome Cell Adhesion Molecule (DSCAM)* as a strong risk factor for ASD. Previous research has shown that *DSCAM* ablation confers social interaction deficits and perseverative behaviors in mouse models. However, it remains unknown to what extent *DSCAM* underexpression captures the full range of behaviors, specifically cognitive phenotypes, presented in ASD. Here, we conducted a comprehensive cognitive behavioral phenotyping which revealed that loss of one copy of *DSCAM*, as in the *DSCAM* ^2J^+/−, that is, *DSCAM* heterozygous mice, displayed hyperactivity, increased anxiety‐like behavior, and motor coordination deficits. Additionally, hippocampal‐dependent learning and memory was affected, including impairments in working memory, long‐term memory, and contextual fear learning. Interestingly, implicit learning processes remained intact. Therefore, *DSCAM* LoF produces autistic‐like behaviors that are similar to those observed in human cases of ASD. These findings further support a role for *DSCAM* dnLoF mutations in ASD and suggest *DSCAM* ^2J^+/− as a suitable model for ASD research.

## Graphical abstract

Autism spectrum disorder represents a growing patient population. Loss of one copy of the *DSCAM* gene in mice reproduces autistic‐like behaviors, such as hyperactivity, motor coordination deficits, and cognitive impairment across several domains. Heterozygous *DSCAM* expression, as in the *DSCAM* ^2J^+/−, represents a promising model for autism research and therapeutic testing.

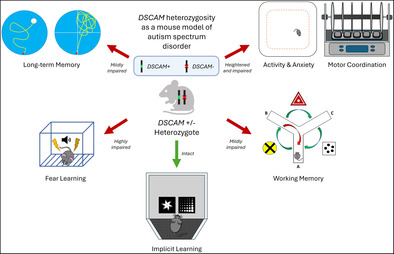

## 1 INTRODUCTION

Over the last decade, autism spectrum disorder (ASD) has been increasing in prevalence considerably, and it is now estimated that 1 in 36 children born in the United States meet the diagnostic criteria for the disorder.^1^ Autism spectrum disorder is characterized by an array of symptoms including somatosensory abnormalities as well as deficits in several social and cognitive domains. Specifically, children and adults diagnosed with ASD can exhibit hyperactivity and heightened anxiety^2^; motor coordination difficulties^3^; social and cognitive impairments, including developmental delays^4^, ^5^, ^6^; and fear learning deficits.^7^ These symptoms often manifest on a spectrum, as the name implies, and therefore affect individuals with differing severity. Furthermore, the causes of most ASD cases are currently unknown. While research had historically focused on environmental circumstances contributing to ASD onset and exacerbation,^8^ the current focus of the field has shifted to genetic risk factors.^9^, ^10^ Indeed, ASD has been found to accumulate within families and appears to be highly heritable.^11^, ^12^ Genome‐wide association studies (GWAS) have found numerous genes to be linked to ASD as risk factors, many of which reside in pathways associated with neurodevelopment and synaptic transmission.^13^, ^14^ One gene that has been repeatedly identified as a significant risk factor of ASD is the *Down Syndrome Cell Adhesion Molecule* (*DSCAM*).^15^, ^16^, ^17^, ^18^, ^19^ Many of the *DSCAM* mutations that have been discovered are de novo loss‐of‐function (dnLoF) mutations,^15^ which likely result in heterozygous *DSCAM* expression. These findings suggest *DSCAM* dnLoF mutations as a potential cause of ASD in the general population.

First characterized in *Drosophila*, *Dscam* is a transmembrane protein that mediates self‐avoidance of the neurites of neurons,^20^, ^21^, ^22^ regulates axonal guidance,^23^, ^24^ and promotes the growth of presynaptic terminals.^25^ In the mouse retina, *DSCAM* determines spatial arrangement of neurons during initial growth and subsequent refinement of projections.^26^, ^27^, ^28^ Moreover, *DSCAM* controls the axon growth of retinal ganglion cells.^29^ Interestingly, the function of *Dscam*/*DSCAM* appears to act in a dose‐dependent manner, as seen in presynaptic growth in both *Drosophila* and mice.^25^, ^30^ This has led *DSCAM* to be studied in the context of multiple neuropsychiatric disorders.^31^ For instance, overexpression of *DSCAM*, which occurs in Down syndrome, has been shown to drive an increase in inhibitory connections in the cortex of Down syndrome mouse models.^30^ On the other hand, a decrease in *DSCAM* expression may underlie some of the key phenotypes that are present in ASD. Dysregulation of NMDA receptors were detected in neurons derived from human iPSCs from an ASD patient where *DSCAM* was deficient, and proper NMDA receptor behavior was restored following the introduction of wild‐type *DSCAM* expression.^32^ Complete knockout of *DSCAM* in neurons and astrocytes in mice accelerates dendritic spine development, which may affect initial synapse formation and maturation during post‐natal growth.^33^ Moreover, *DSCAM* knockout mice appear to replicate some of the repetitive behaviors and social impairments that are associated with ASD, further promoting *DSCAM* LoF mutations as a potential cause of ASD.^33^ However, ASD is often caused by heterozygosity of risk loci as opposed to a complete ablation of gene expression.^15^, ^34^ Indeed, neural stem cell‐specific *DSCAM* heterozygous mice also display significant deficits in sociability measures.^32^ Whether *DSCAM* heterozygous mice exhibit the full range of ASD‐like behavioral phenotypes, particularly those related to cognitive paradigms, is an important question and is currently unknown.

Here, we aimed to provide a comprehensive behavioral assessment of *DSCAM* heterozygous mice (*DSCAM* ^2J^+/−, i.e., heterozygotes) that would determine the value of this mouse model for ASD research. We focused on murine behaviors that correlated with those described in ASD, namely activity and sensorimotor function, working and long‐term memory, fear learning, and procedural learning. We found that *DSCAM* heterozygosity because of a LoF mutation was sufficient to drive hyperactivity and notable motor coordination deficits along with minor impairments in spatial learning and memory. Additionally, these mice show a substantial and lasting deficit in contextual fear learning. Our findings support a decrease in *DSCAM* expression as a possible explanation of ASD‐related behaviors and provide strong evidence for a disease model that could be useful for ASD research and therapeutic testing.

## 2 MATERIALS AND METHODS

### 2.1 Animals, breeding, and genotyping

For all experiments, littermates were age‐matched and divided into cohorts (Table 1). The *DSCAM* ^2J^ (C3H/HeDiSn‐*DSCAM* ^*2J*^/GrsrJ) mice were originally acquired from Jackson Laboratories (Strain #: 006038) and migrated to a C3H background by crossing *DSCAM* ^2J^+/− mice to sighted C3H (C3Sn.BLiA‐*Pde6b* ^+^/DnJ), also acquired from Jackson Laboratories (Strain #: 003648), for 10+ generations. *DSCAM* ^2J^ were then maintained by the same cross to produce heterozygous (+/−) and wild‐type (+/+) littermates. Therefore, *DSCAM* ^2J^+/− will also be referred to as heterozygotes and *DSCAM* ^2J^+/+ as wild‐type. The *DSCAM* ^2J^ mutation was identified at Jackson Laboratories and characterized at the University of Idaho.^26^ It results in a four base pair duplication that leads to a frameshift mutation and subsequent truncation of the protein, which is similar to many of the dnLoF mutation variants that have been identified in individuals with ASD.^15^, ^19^, ^35^

**TABLE 1:** Summary of cohorts and respective behavioral tests. All cohorts were composed of littermates resulting from a mating cross between *DSCAM* ^2J^+/− and C3H. Mice were age‐matched and entered behavioral testing at 2–5 months of age. Behavioral tasks were organized according to degree of aversiveness, with any task that included an electrical foot‐shock being run as the terminal experiment. Cohorts that participated in multiple experiments were given at least 1 week rest between successive tasks.

Cohort no.	Experiments	Number of Wild‐types	Number of Heterozygotes
Cohort 1	Open field exploration Morris water maze Fear conditioning	8 (4 M, 4 F)	16 (7 M, 9 F)
Cohort 2	Y‐maze Passive avoidance	12 (7 M, 5 F)	12 (4 M, 8 F)
Cohort 3	Touchscreen pretraining Visual discrimination	17 (10 M, 7 F)	12 (5 M, 7 F)
Cohort 4	Rotarod Hot plate	9 (4 M, 5 F)	7 (2 M, 5 F)

Mice were group housed by sex, at a maximum of 5 animals per cage, in a temperature‐controlled vivarium (22°C) with a 14/10‐h light/dark cycle until they had reached the designated age range to begin studies. Mice were provided ad libitum food and water, except during visual discrimination experiments as detailed. All mice were 2–6 months of age during the experiments and both males and females were used.

All genotyping was performed on tail tips by Transnetyx (Memphis, TN) based on protocols developed by Jackson Laboratories. All mouse work and protocols were approved by the University of Michigan Institutional Animal Care and Use Committee (IACUC) and in accordance with the National Research Council Guide for the Care and Use of Laboratory Animals (NIH).

### 2.2 Behavioral tests

All experiments were conducted with the investigator blind to genotype and were performed during the light phase of the light/dark cycle, usually between 8:00 a.m. and 4:00 p.m. Cohort sample sizes were determined based on need of the subsequent experiments, and estimates were calculated with a detection level of ~10% using G*Power software^36^ with *α* set to 0.05 and the power (1−*β*) value set to 0.95. Standard deviation values were derived from historical lab data on the included behavioral tasks. Sex was considered as an independent variable and determined to be underpowered for direct assessment.

#### 2.2.1 Open‐field exploration

To assess exploratory behavior, including activity levels, open‐field experiments were conducted. Experiments were carried out in a rectangular open arena (74 × 74 × 29 cm) with smooth white opaque walls and floor made of acrylic. The arena was illuminated at approximately 45 lumens, measured at the center. Each mouse was individually placed into the center of the arena and allowed to freely explore the environment for 10 min, after which the mouse was removed and returned to their home cage. Between each mouse the arena was cleaned with 70% ethanol. LimeLight 3 video tracking software (Actimetrics, Evanston, IL) and a camera mounted above the arena were used to record behavior during the trial. Total distance traveled, time spent in the inner zone of the arena (46 × 46 cm), and the number of crossings between zones was analyzed offline. One mouse was removed because of inactivity that was statistically determined to be an outlier.

#### 2.2.2 Rotarod

Locomotor coordination and activity were evaluated using the rotarod (TSE Systems, Chesterfield, MO). Mice were placed on the rotating rod, initially moving at a constant rate of 2 rpm. Following a 10 s habituation period to allow the mice to get accustomed to initial rotation, the rod was then accelerated from 4 to 40 rpm at a constant rate. The maximum trial duration was set to 5 min. Mice were run one at a time and given a single trial each day for three consecutive days. Latency to fall was recorded by the TSE RotaRod software using the pressure plate in the flooring of the apparatus and manually by the experimenter as a backup.

#### 2.2.3 Hot plate

Sensory response to thermal nociception, that is, pain sensitivity, was measured using the hot plate test (IITC Life Sciences, Woodland Hills, CA). The black aluminum surface (27 × 30 cm) was maintained at 50 ± 0.5°C and confirmed to be stable using a thermistor probe connected to the hot plate apparatus. An acrylic cylindrical enclosure (15 cm height, 10 cm inner diameter) was used to limit the movement of the mice on the heated surface during the experiment. Each mouse was placed onto the surface and allowed to explore while being closely monitored by an observer. The latency to hindlimb lick was determined and recorded as the response time, at which point the mouse was promptly removed from the enclosure and returned to their home cage.

#### 2.2.4 Y‐maze

Working memory was measured by spontaneous alternation in the Y‐maze. The Y‐maze was built with three symmetrical arms (7.5 × 18 × 13 cm) at a 120° angle. The maze was constructed of rigid white opaque acrylic. Connecting the three arms is an equilateral triangular space (7.5 cm). Each trial began with the mouse being placed in the center triangular space facing the same arm. The mouse was then allowed to explore the maze for 8 min. An arm entry was recorded when the center‐point of the mouse crossed the half‐way point of the arm. Triads were recorded, which is defined as a set of three arm entries. A correct triad was determined to be consecutive entries into the three different arms of the maze, and any other combination was considered an error. The percent alternation calculation is presented below. Direct revisits were recorded when a mouse leaves an arm, enters the center zone, and returns to the same arm again. Indirect revisits were defined as when a mouse leaves an arm, passes through the center zone, enters another arm, and then returns to the original arm. Behavior was recorded using an overhead camera and analyzed through EthoVision XT v17 software (Noldus, Wageningen, the Netherlands).

$$\text{Percent Alternation}=\frac{\text{Number of correct triad alternations}}{\text{Total number of}\;\mathrm{arm}\;\text{entries}-2}\times 100$$

#### 2.2.5 Morris water maze

Long‐term spatial learning and memory was assessed using the Morris water maze (MWM). The arena was a 1.2‐m diameter pool filled with water that was semi‐opaque through the addition of white nontoxic paint. The water temperature was maintained at 25 ± 2°C using an electric heating mat underneath the pool. For each of the training trials, a circular platform (10 cm diameter) was placed in the NE quadrant of the pool and sat approximately 1 cm below the water surface. Each training session began with the mouse being placed on the platform for ~10 s before starting their first trial. The mouse was then placed into the water along the wall and allowed to search the arena for the platform. The training trial ended when the mouse reached the platform or when 60 s had elapsed. Mice were trained for four trials per day for 9 consecutive days, and the starting position for each trial was chosen pseudo‐randomly from seven potential positions. On training days 4, 7, and 10, the mice were evaluated in a probe trial that was conducted before any training trials on the day. The probe trial removed the platform from the pool to assess their memory of the platform location. Mice were placed opposite the platform's usual location and given a 60 s trial to search the arena. On the last day of the experiment and following the final probe trial, visible platform trials were conducted to assess locomotor and visual capabilities. The visible trials consisted of six trials where the platform now had a visual cue (a paper flag) to mark the platform location. Trials were run in sets of 2, where the platform was changed to a different quadrant for each set. Training trial duration was averaged together for each day to represent that mouse's performance, where applicable. Behavior was recorded by an overhead camera at 15 fps and analyzed through the WaterMaze software (Actimetrics, Evanston, IL).

#### 2.2.6 Fear conditioning

Contextual and cue‐based fear conditioning was conducted to assess long‐term fear learning and memory. Four operant chambers (Med Associates, Fairfax, VT) were organized on a steel rack in an isolated room. The chamber was constructed of clear acrylic on the front, back, and top, and aluminum on the two remaining sides. The floor was made of stainless‐steel parallel bars forming a grid with a metal tray positioned below. Each of the metal floor grids was connected to a shock controller, which was managed by a desktop computer running FreezeFrame software (Actimetrics, Evanston, IL). A camera was positioned above each chamber to record mouse behavior back to the same computer. The primary measure was freezing, which was assessed using the FreezeFrame software to compare the movement of the mouse frame by frame.

The surrounding environment was altered depending on the chosen context. Two contexts were used throughout the experiments, designated Context A and Context B. Context A consisted of white room lighting, white noise from a sound machine, 70% ethanol as a cleanser and an odor, and a floral‐patterned curtain that covers the chambers during the session. Context A was used for the training and context recall components of the experiment. Context B was significantly modified to act as a novel environment in comparison to Context A. Therefore, Context B used red room lighting, no added noise, 2% acetic acid as a cleanser and odor, and a blue and white striped curtain. Additionally, the metal floor grid was covered with a white acrylic sheet and a cushioned mat flooring, and a curved sheet of white acrylic was inserted into the chamber to alter its dimensions.

Using the four chambers, mice were transferred from their home cages to the chambers individually. The experiment was conducted on consecutive days, with the first 3 days acting as conditioning training. For conditioning sessions, mice were placed into Context A for 3 min prior to a 30 s tone (2.8 kHz, 75 dB) whose termination aligned with a foot‐shock (2 s, 0.75 mA). Thirty seconds later, the mice were removed from the chamber. To test contextual memory, 24 h after the final conditioning session the mice were returned to Context A for a 5‐min period in the absence of any tone or foot‐shock. Finally, to test cued memory, 24 h after the context test the mice were placed in Context B for 3 min, after which the 30 s tone was initiated three times with a 30 s inter‐tone interval. Mice were then returned to their home cage.

During training, one mouse escaped from the chamber and was pursued by the investigator until caught. This mouse was subsequently removed from the analysis because of a distinct experience that could be fear inducing, which follows standard exclusion criteria for this behavioral task.

#### 2.2.7 Passive avoidance

Fear learning, in which the animal must choose between approach and avoidance, was evaluated using the Passive Avoidance paradigm. The apparatus (Maze Engineers, Shokie, IL) consisted of two chambers (20 × 20 × 20 cm) that were connected via an automated sliding door (7 × 5 cm). The chambers were constructed of black acrylic walls, a clear acrylic lid, and stainless‐steel grid floor. One chamber was well‐lit, with the lid being kept clear, and a bright light was present within the chamber to invoke photophobia. The second chamber was kept dark, in which the lid was shrouded in black construction paper and the light was not turned on. During the habituation phase, the mouse was introduced to the light chamber and allowed to explore for 1 min, at which point the sliding door was opened to reveal access to the dark chamber. The latency to cross was recorded via infrared beams, and the door was closed once the mouse crossed over to the dark chamber. After an additional minute of exploration of the dark chamber, the lid was removed, and the mouse was returned to their home cage. The maximum time to cross was set to 10 min. Each mouse was given three such habituation trials on the first day of the experiment. On the second day, referred to as the acquisition trial, the mouse is again allowed to cross to the dark chamber. After 1 min an aversive stimulus, a mild foot‐shock (3 s, 0.4 mA), was delivered through the floor grid and the mouse was removed from the chamber 30 s after the foot‐shock. Twenty‐four hours after the foot‐shock was delivered, the mouse was placed into the apparatus again, and the latency to cross to the dark chamber was recorded in the Passive Avoidance software. A longer latency is correlated with a stronger association of the dark chamber with the aversive stimulus.

#### 2.2.8 Touchscreen and visual discrimination

Procedural learning and higher‐level discriminatory learning was assessed using the Bussey‐Saksida Touchscreen system (Lafayette Instrument, Lafayette, IN). To avoid neophobia, the liquid reward, strawberry Ensure™ milkshake, was introduced to the home cage ad libitum for 3 days prior to the experiment beginning. Before the mice could be tested in visual discrimination, they were taken through a protocol to become adjusted to the touchscreens. However, to provide additional motivation, the mice were food deprived to 90%–95% of their body weight. The mice were then accustomed to the touchscreen chamber environment for the first time in a 10‐min session. Habituation continued for three additional days, with autotraining to the strawberry milkshake reward (20 μL) in 20 and then two 40‐min sessions. To begin the pretraining phase, mice were then trained to Initial Touch, where images were randomly displayed on the screen and an interaction with the screen elicited 3× the reward (60 μL) and initiated a 10 s inter‐trial interval (ITI). The Initial Touch phase lasted 3 days to encourage association between screen interaction and reward. Each mouse then began the Must Touch phase, where interaction with a displayed image was required to elicit a reward, while interaction with a blank screen incurred no penalty. Completing 30 trials within a 1‐h period satisfied this phase and advanced the mouse to the Must Initiate phase. Within this phase, mice must first nose‐poke to initiate a trial and bring an image onto the screen and then interact with that image for a reward. Successful completion of 30 trials within 1‐h progressed the mice to the Punish Incorrect phase, which introduced a 20 s timeout penalty for interaction with a blank image location. This phase required a criteria of 24/30 correct trials, or 80%, to complete the pretraining and advance to visual discrimination. All mice progressed through the phases depending on their individual metrics, and therefore their days and trials to criterion/completion were recorded as a measure of their performance. Two of the mice did not complete the pretraining steps, remaining sedentary during the majority of the sessions, and were therefore removed from the study.

Once pretraining was complete, mice began 2‐choice visual discrimination. Two images were displayed at once, interaction with the one image was rewarded (S+) and reinforced with strawberry milkshake reward while the other (S−) was punished with a 20 s timeout. Interaction with the incorrect image instituted correction trials, where the trial was repeated until the S+ was chosen successfully. Training was continued on weekdays until criterion was reached, which was set at 21/30 correct trials, or 70%, and maintained for 2 consecutive days. Mice were limited to a total of 40 days to complete the visual discrimination task, and any mice that did not complete the task within that timeframe were removed from the analysis. Therefore, 6 wild‐type and 4 heterozygotes were removed.

### 2.3 Statistical analysis

All experiments and analyses were performed with the experimenter blind to genotype. Analysis of the data was performed using GraphPad Prism 10. Average data is presented in the figures as the mean ± the standard error of the mean (SEM). The appropriate statistical tests were chosen depending on the experiment and are indicated as such within the figure legends. These tests include: Two‐tailed unpaired Student's *t*‐test (for comparison of groups); Mixed‐effects model (REML); Log‐rank (Mantel‐Cox) test; Two‐way ANOVA + planned post‐hoc comparisons with Bonferroni correction and/or repeated measures (for multifactor comparisons and repeated timepoints); and One‐tailed Student's *t*‐tests (for comparison against chance). Statistical significance was considered as *p* < 0.05.

## 3 RESULTS

In order to conduct a thorough behavioral phenotyping, cohorts were taken through a series of behavioral tasks and tests. Multiple cohorts were used throughout the study and evaluated in different sets of experiments. The composition of each cohort and which tests they were subjected to are summarized in Table 1.

### 3.1 Loss of one copy of *DSCAM* heightens the state of anxiety and promotes hyperactivity

**FIGURE 1:**
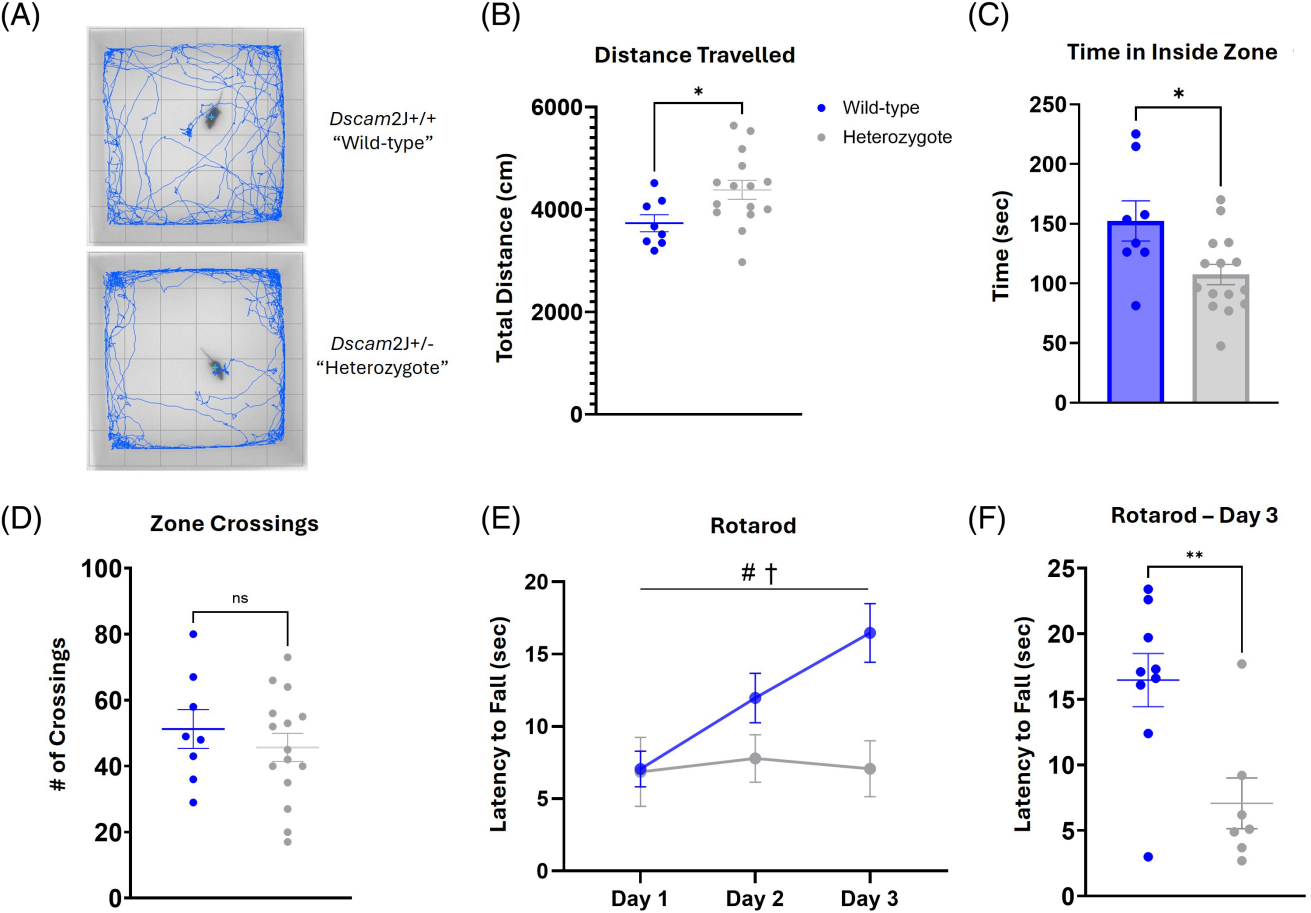
*DSCAM* ^2J^+/− heterozygotes exhibit hyperactivity, heightened anxiety, and impaired motor coordination. Exploratory behavior was assessed in the open field during a 10‐min trial. (A) Representative trajectories of individual animals demonstrate the discrepancy between genotypes. (B) Heterozygotes traveled significantly more distance during exploration. (C, D) Heterozygotes spend less time in the inner zone but there was no significant difference in the number of crossings between zones. Locomotor coordination was evaluated on an accelerating rotarod. (E, F) Latency to fall was measured, and found to be significantly less in heterozygotes. Results are presented as Mean ± SEM. (A–D) Open field *DSCAM* ^2J^+/− *n* = 15 and WT *n* = 8. Rotarod *DSCAM* ^2J^+/− *n* = 7 and WT *n* = 9. Statistical tests are as follows: (B–D, F) Two‐tailed *t*‐test. (E) Mixed‐effects model (REML) where # denotes main effect of time and † denotes main effect of genotype. Significant differences are shown (*p < 0.05 and **p < 0.01).

Activity levels in a novel environment were assessed in the open‐field exploration task. Exploration and activity were determined as the total distance traveled during the 10‐min test period, while anxiety was measured by the willingness to explore and remain in the inner zone of the arena. Representative exploration paths are presented in Figure 1A. When compared with the wild‐type controls, the heterozygotes showed increased distance traveled during the test period (Figure 1B; *t* = 2.310, *df* = 21, *p* = 0.0312). Additionally, the time spent in the inner zone of the arena was decreased in the heterozygotes (Figure 1C; *t* = 2.667, *df* = 21, *p* = 0.0144) signaling a possible heightened state of anxiety present. There was no difference observed between the genotypes in the number of crossings between the zones (Figure 1D; *t* = 0.7716, *df* = 21, *p* = 0.4489). Altogether, these results suggest that the heterozygotes are hyperactive and equally motivated to explore the novel environment as the wild‐type mice, as seen by similar number of zone crossings, but that they do not spend prolonged periods of time in the inner zone because of increased anxiety‐like behavior.

### 3.2 *DSCAM* heterozygotes exhibit impaired locomotor coordination without affecting nociception

As the *DSCAM* ^2J^ null has been previously observed to have an altered gait^26^ that could affect their motor performance, locomotor coordination and motor learning was assessed in the *DSCAM* ^2J^+/− using the rotarod balancing task. To perform the task, it is necessary that the mice shift and adjust their balance on the rod as it slowly accelerates, and their latency to fall off the rod is recorded. This task was repeated for three consecutive days to allow the mice to learn the task and improve their latency. Unlike the wild‐type controls, the heterozygotes showed no improvement across time (Figure 1E; effect of time *F* (1.552, 20.96) = 5.152, *p* = 0.02; effect of genotype *F* (1, 14) = 5.214, *p* = 0.039). This was further discovered through an ad‐hoc comparison of performance on Day 3 of training, where the heterozygotes had a decreased latency to fall compared with the wild‐type (Figure 1F; *t* = 3.279, *df* = 14, *p* = 0.0055), suggesting an impairment in motor coordination.

To examine sensory efficiency, specifically through thermal nociception, the hot plate test was conducted. This test determined the nocifensive behavior response time of each mouse to a heated surface, which was then used as a proxy for the peripheral pain response. There was no difference between the groups in latency to hindlimb lick, indicating that loss of one copy of *DSCAM* produces no overt alterations in thermal nociception (Figure S1A; *t* = 0.4083, *df* = 14, *p* = 0.6892).

### 3.3 Impaired working memory in *DSCAM* heterozygotes

**FIGURE 2:**
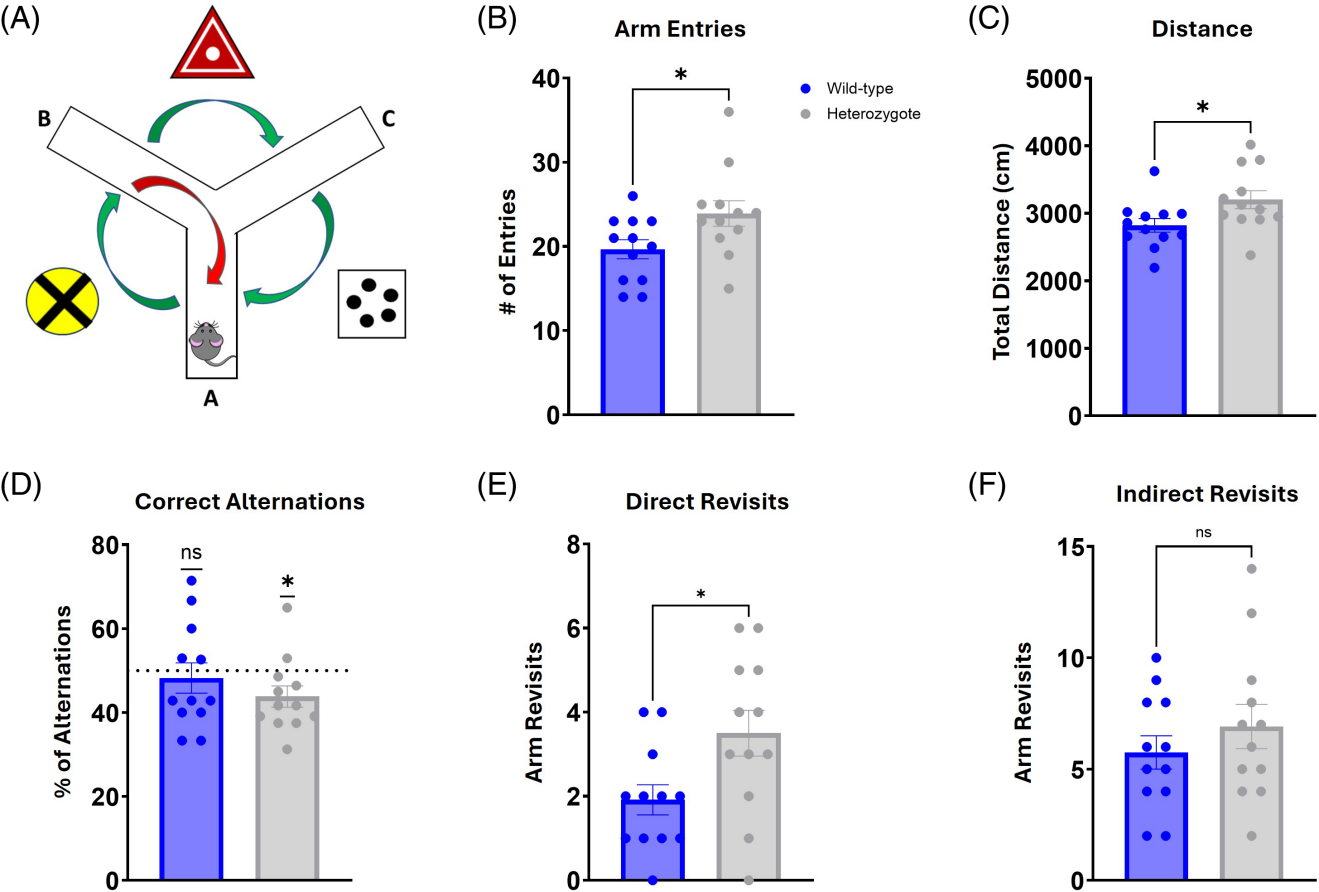
*DSCAM* ^2J^+/− heterozygotes are hyperactive and show impairments in spatial working memory. Short‐term learning and memory were probed in 8‐min trials in the Y‐maze. (A) Representation of the maze layout, with three visual cues across from each arm. Correct spontaneous alternations (green arrows) compared with alternation errors (red arrow) were considered as a metric of working memory during maze exploration. (B, C) Heterozygotes completed significantly more entries into arms and traveled further during the trial. (D) Percentage of triads registered as a correct alternation was significantly below chance (dotted line = 50%) for heterozygotes. (E, F) Direct revisit errors were significantly greater in heterozygotes, while indirect revisit errors were shown to be not different between the groups. Results are presented as Mean ± SEM. Sample size: *DSCAM* ^2J^+/− *n* = 12 and WT *n* = 12. Comparison between heterozygotes and WT mice was analyzed by a two‐tailed *t*‐test, or a one‐tailed *t*‐test calculated against chance (50%), and the significant differences are shown (*p < 0.05 and **p < 0.01).

The Y‐maze was used to assess spontaneous alternation as a measure of spatial working memory using visual cues (Figure 2A). As we had previously observed in the open‐field exploration task, the heterozygotes displayed hyperactivity in their 8‐min exploration of the Y‐maze as well. When compared with the wild‐type controls, the heterozygotes had more arm entries (Figure 2B; *t* = 2.249, *df* = 22, *p* = 0.0349) and traveled a greater distance (Figure 2C; *t* = 2.279, *df* = 22, *p* = 0.0327). In alternation rate, the heterozygotes completed fewer correct alternations than chance (50%) would have expected (Figure 2D; wild‐type *t* = 0.4863, *df* = 11, *p* = 0.6363; heterozygote *t* = 2.432, *df* = 11, *p* = 0.0333), indicating a greater number of errors and a deficit in spontaneous alternation. Those errors were represented by an increase in direct revisits when compared with wild‐type controls (Figure 2E; *t* = 2.433, *df* = 22, *p* = 0.0236). Indirect revisit errors were similar between the groups (Figure 2F; *t* = 0.9358, *df* = 22, *p* = 0.3595). These findings indicate a significant impairment in the short‐term, or working, memory of the *DSCAM* heterozygous mice.

### 3.4 Loss of one copy of *DSCAM* impairs memory recall in the Morris water maze task

**FIGURE 3:**
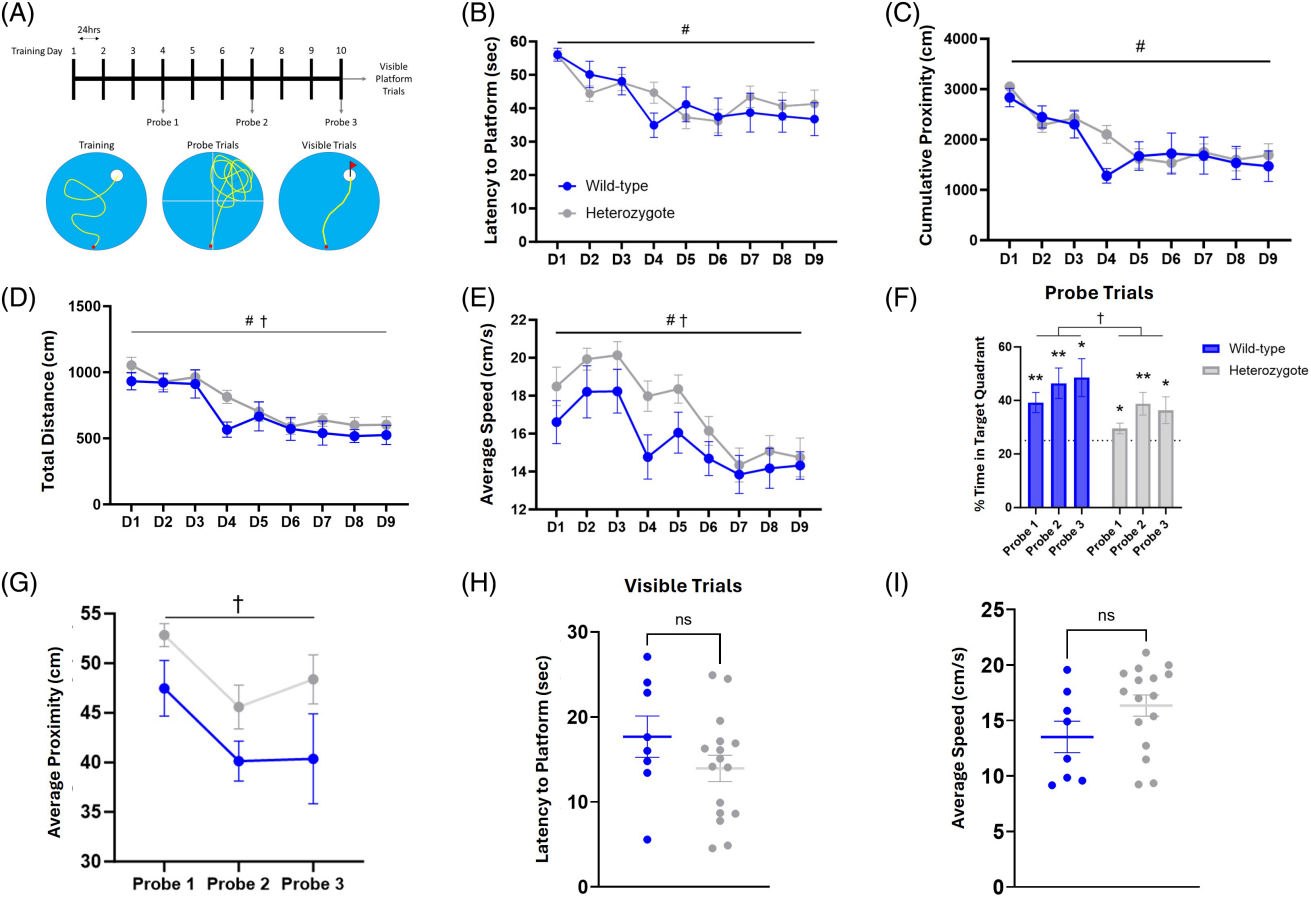
*DSCAM* ^2J^+/− heterozygotes display spatial memory deficits in the Morris water maze (MWM). (A) Training consisted of nine consecutive days of four trials per day in which mice were allowed to explore for 60 s or until they reached the hidden platform, which is set just below the surface of the water. (B) During training, the latency to reach the platform significantly decreased across days with no significant differences between groups. (C) The cumulative proximity, which is a measure of proximity to the platform location during each trial, also decreased with training days but resulted in no difference between groups. (D, E) While there was no difference between the groups in respect to distance traveled during training, the average speed was significantly increased in the heterozygotes. Three probe trials were given to test memory of the platform location. During these trials, the platform was removed from the pool and the mice were given 60 s to search for the now removed platform, measuring the percentage of time spent searching in the correct quadrant of the pool as a measure of spatial memory. (F) Both groups performed significantly better than chance in all three probe trials, but WT outperformed heterozygotes across probes. (G) The average proximity to the correct platform location was significantly increased in heterozygotes. To ensure there were no other behavioral factors present in the groups that might impact performance, on Day 10 the mice received six trials where the platform was highlighted by a visible flag. (H, I) Both groups were able to quickly swim to the platform, showing no significant differences in visual recognition or swim speed. Results are presented as Mean ± SEM. Sample size: *DSCAM* ^2J^+/− *n* = 16 and WT *n* = 8. Statistical tests are as follows: (B–E) Two‐way ANOVA, where # denotes main effect of training and † denotes main effect of genotype. (F) One‐sample *t*‐test compared with chance = 25% and two‐way ANOVA where † marks main effect of genotype. (G) Two‐way ANOVA, where † denotes main effect of genotype. (H, I) Two‐tailed *t*‐test. Significant differences are shown (*p < 0.05 and **p < 0.01).

The Morris water maze tests spatial learning and memory based on environmental cues. The 10‐day protocol consists of 9 training days, 3 inter‐training memory probes, and visible trials on Day 10 (Figure 3A). Both groups learned the task and improved their training performance across time, with no difference between groups (Figure 3B; effect of time *F* (5.680, 125.0) = 7.829, *p* < 0.0001; effect of genotype *F* (1, 22) = 0.1018, *p* = 0.7527). Similarly, both groups decreased their cumulative proximity across training days, to the same extent (Figure 3C; effect of time *F* (8, 198) = 9.188, *p* < 0.0001; effect of genotype *F* (1, 198) = 1.339, *p* = 0.2486). Heterozygotes showed a significant increase in distance traveled during training (Figure 3D; effect of time *F* (8, 198) = 12.61, *p* < 0.0001; effect of genotype *F* (1, 198) = 5.946, *p* = 0.0156) and displayed greater swim speed (Figure 3E; effect of time *F* (8, 198) = 7.604, *p* < 0.0001; effect of genotype *F* (1, 198) = 11.63, *p* = 0.0008).

To test their memory recall, the mice were evaluated three times throughout the training procedure in probe trials. Compared against chance (25%), both groups showed a preference for exploring the target quadrant (Figure 3F; wild‐type P1 *t* = 3.810, *df* = 7, *p* = 0.0066; wild‐type P2 *t* = 3.769, *df* = 7, *p* = 0.007, wild‐type P3 *t* = 3.324, *df* = 7, *p* = 0.0127; heterozygote P1 *t* = 2.267, *df* = 15, *p* = 0.0386; heterozygote P2 *t* = 3.252, *df* = 15, *p* = 0.0054; heterozygote P3 *t* = 2.258, *df* = 15, *p* = 0.0393). However, when the groups were compared against each other the heterozygotes display significantly poorer performance (Figure 3F; effect of genotype *F* (1, 22) = 4.625, *p* = 0.0428). Furthermore, heterozygotes showed an increase in their average proximity to the platform location during the probe trials (Figure 3G; effect of probe *F* (1.890, 41.59) = 5.191, *p* = 0.0108; effect of genotype *F* (1, 22) = 6.584, *p* = 0.0176). These results suggest a moderate deficit in spatial learning and memory in the *DSCAM* heterozygotes.

To determine whether there were any visual or locomotor differences that could serve as confounds, on the final day of the experiment the visual platform trials were run. The heterozygotes showed no significant difference in latency to find the platform compared with wild‐type controls (Figure 3H; *t* = 1.343, *df* = 22, *p* = 0.1931) nor did they display a significant difference in swim speed (Figure 3I; *t* = 1.690, *df* = 22, *p* = 0.1051). These results demonstrate that the poor performance exhibited by the heterozygotes was not because of visual or locomotor deficits.

### 3.5 Loss of one copy of *DSCAM* impairs Pavlovian fear learning

**FIGURE 4:**
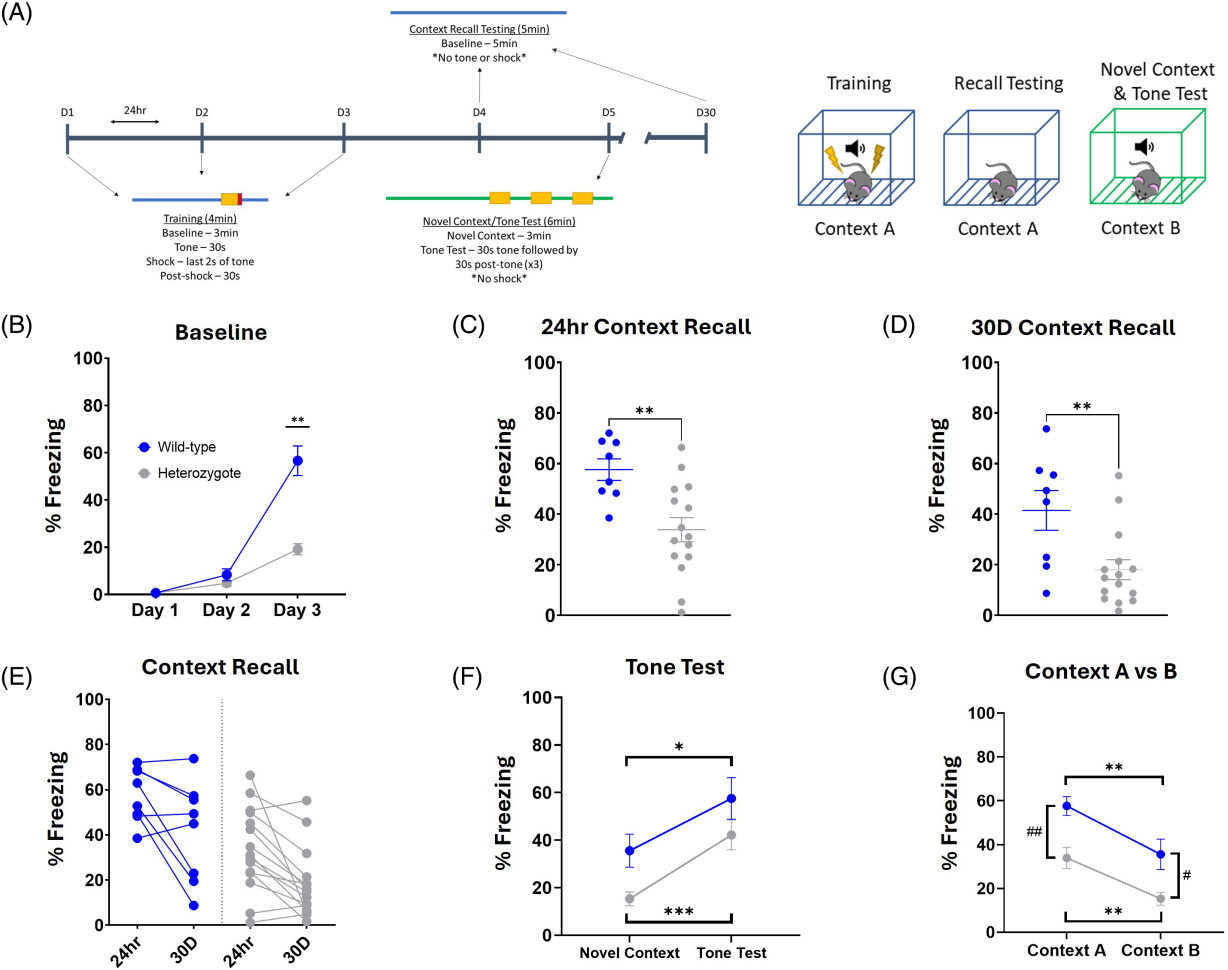
*DSCAM* ^2J^+/− heterozygotes exhibit long‐lasting deficits in contextual fear learning and memory. (A) For 3 days, mice were placed in a training context (Context A) and allowed to explore for 180 s before a 30 s tone is played that co‐terminated with a foot‐shock (2 s, 0.75 mA). Following training, contextual recall was carried out 24 h later in the same context (Context A). To test tone recall, on Day 5 mice were introduced to a completely novel context (Context B), allowed to explore for 180 s after which the 30 s tone is repeated 3× with 30 s inter‐tone intervals. Thirty days after the last training day, mice were reintroduced to the training context (Context A) to be tested for extended contextual recall. (B) During training, heterozygotes displayed significant deficits in fear learning, measured by percent freezing that became evident by the third training day. (C) Heterozygotes exhibited significantly less freezing during the contextual recall. (D, E) Furthermore, the deficit in fear learning and memory was present 30 days later, as many of the heterozygotes showed substantial decrement in freezing during the 30‐day contextual recall. (F) Both groups displayed intact tone recall in the novel context/tone test on Day 5. (G) Likewise, both groups exhibited a significant reduction in freezing levels in the novel context (Context B). Results are presented as Mean ± SEM. Sample size: *DSCAM* ^2J^+/− *n* = 15 and WT *n* = 8. Comparison between heterozygotes and WT mice was analyzed by a two‐way ANOVA with planned post‐hoc comparisons and/or two‐tailed *t*‐test. (G) Two‐way ANOVA, where * denotes an effect of test and # denotes an effect of genotype. Significant differences are shown (* and #*p* < 0.05, ** and ##*p* < 0.01, ****p = 0.0001).

Tone‐cued and contextual fear conditioning were used to assess Pavlovian fear learning and memory. The 5‐day protocol includes 3 days of tone‐shock paired training, contextual recall 24 h later, tone response testing in a novel context on Day 5, and a final contextual recall at 30 days (Figure 4A). During training days, mice experience a 3‐min baseline period that can be used to establish a learning curve. While both groups did display learning, heterozygotes showed a significant deficit in baseline freezing (Figure 4B; effect of time *F* (1.306, 27.42) = 163.6, *p* < 0.0001; effect of genotype *F* (1, 21) = 32.74, *p* < 0.0001; post‐hoc comparison of days D1 *p* > 0.9999, D2 *p* = 0.7074, D3 *p* = 0.0010). This freezing deficit in the heterozygotes was present in the 24‐h context recall test (Figure 4C; *t* = 3.254, *df* = 21, *p* = 0.0038). Thirty days after the final training day, mice were subjected to a context recall test again, where heterozygotes still displayed a deficit in contextual fear recall (Figure 4D; *t* = 2.989, *df* = 21, *p* = 0.007). A comparison of the 24‐h and 30‐day context recall showed heterozygotes had a substantial reduction in freezing at the level of the individual subject (Figure 4E), suggesting a lack of long‐term consolidation of the fear memory. In tone‐cued fear conditioning in a novel context both groups exhibited a freezing response to the tone (Figure 4F; effect of test *F* (1, 21) = 26.15, *p* < 0.0001; wild‐type *p* = 0.0192, heterozygote *p* = 0.0002) but the tone responses were not statistically different between groups (Figure 4F; post‐hoc comparison of tone test *p* = 0.1798). Both groups showed a significant reduction in freezing behavior when introduced to a novel context (Figure 4G; effect of context *F* (1, 21) = 24.06, *p* < 0.0001; wild‐type *p* = 0.0069, heterozygote *p* = 0.0021) but wild‐type mice exhibited a heightened generalization in the novel context (Figure 4G; effect of genotype *F* (1, 21) = 16.11, *p* = 0.0006; Context B *p* = 0.0104). Overall, these results demonstrate a considerable contextual fear learning deficit in the heterozygotes.

**FIGURE 5:**
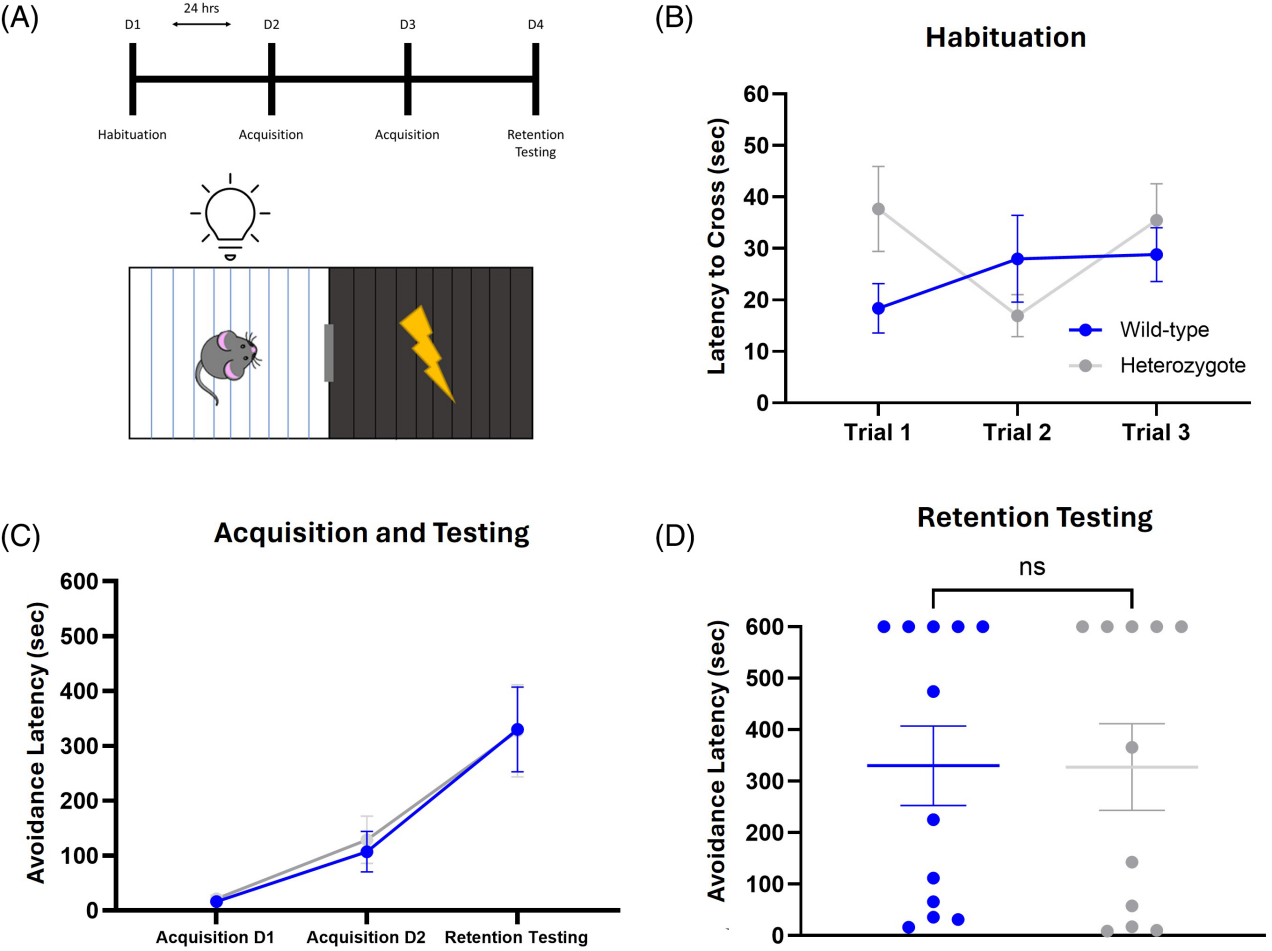
*DSCAM* ^2J^+/− heterozygotes do not display deficits in fear‐motivated decision‐making. (A) On the first day, mice were allowed to explore and habituate to both the illuminated and dark chamber. Following habituation, 2 days of acquisition were completed in which mice were subjected to an unsignaled foot‐shock (3 s, 0.4 mA) after crossing into the dark chamber. Retention testing was performed 24 h later. The latency to cross, or the avoidance latency, was recorded as a measure of willingness to enter the dark chamber. (B) Both groups were willing to cross into the dark chamber and did so equally across three habituation trials. (C, D) During acquisition and retention testing, both groups exhibited an increase in avoidance latency but there were no differences between the groups. Results are presented as Mean ± SEM. Sample size: *DSCAM* ^2J^+/− *n* = 11 and WT *n* = 12. Comparison between heterozygotes and WT mice was analyzed by a two‐way ANOVA with planned post‐hoc comparisons and/or two‐tailed *t*‐test.

To examine an alternative form of fear learning that integrates fear‐motivated decision‐making, mice were evaluated in the passive avoidance task. This task incorporates associative learning and the suppression of the innate desire to enter the dark environment to avoid an aversive stimulus. The 4‐day protocol begins with habituation to the chambers followed by 2 days of acquisition and ends with a retention testing session (Figure 5A). During habituation, mice were given three trials to acclimate to the chambers. Importantly, there was no inherent difference between the groups in their willingness to cross over to the dark chamber once the dividing door opens (Figure 5B; effect of genotype *F* (1, 21) = 0.6785, *p* = 0.4194). However, there was also no notable difference between the groups in the acquisition training (Figure 5C; effect of genotype *F* (1, 21) = 0.02969, *p* = 0.8648) or in the 24 h retention phase (Figure 5D; *t* = 0.02217, *df* = 21, *p* = 0.9825). These results indicate that the heterozygotes are capable of integrating an instrumental response (i.e., avoidance) with Pavlovian aversive conditioning to the same extent as wild‐type mice.

### 3.6 Visual procedural learning is intact in *DSCAM* heterozygotes

**FIGURE 6:**
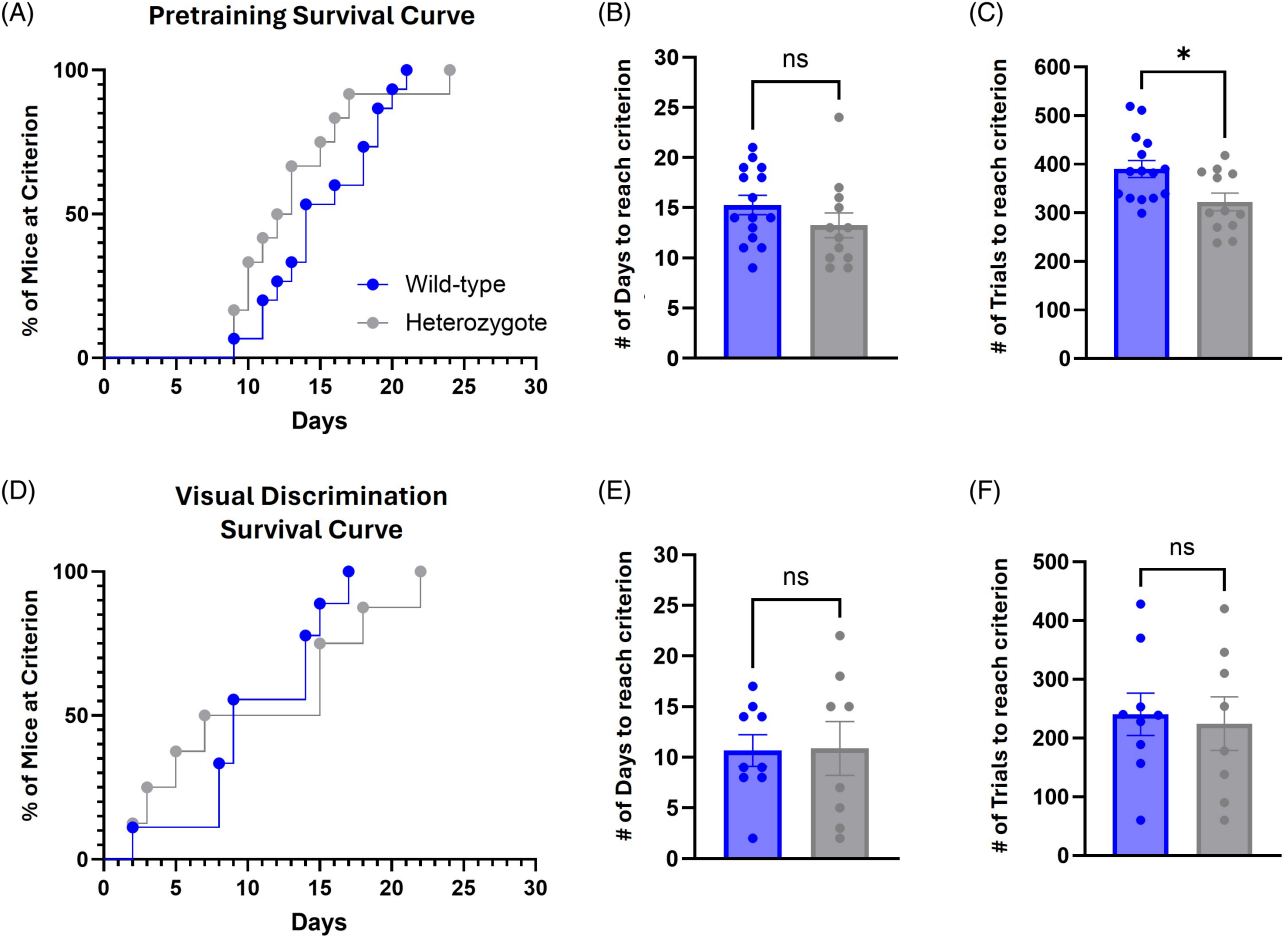
*DSCAM* ^2J^+/− heterozygotes exhibit normal procedural learning and higher‐level discriminatory learning. Procedural learning and visual discrimination were assessed in Bussey‐Saksida Touchscreen systems in daily sessions until criterion was reached. (A) Pretraining presented as a survival curve indicated no gross differences between groups in the number of days to reach criterion. (B, C) Heterozygotes and WT mice required similar numbers of days to reach criterion during pretraining but needed less trials within those days to progress. Following pretraining, mice were assessed in a pairwise visual discrimination task. (D) The survival curve for visual discrimination revealed no significant differences in the number of days to reach criterion, set at 70%. (E, F) Similar to pretraining, both groups required nearly the same number of days and trials to complete the task. Results are presented as Mean ± SEM. During pretraining, sample size *DSCAM* ^2J^+/− *n* = 12 and WT *n* = 15. For visual discrimination, several mice in each group did not complete the task within the given timeframe and were removed from the analysis, leading the sample size to be reduced to *DSCAM* ^2J^+/− *n* = 8 and WT *n* = 9. Comparison between heterozygotes and WT mice was analyzed by a Log‐rank (Mantel‐Cox) test (A and D) or two‐tailed *t*‐test.

The Bussey‐Saksida Touch Systems were used to evaluate complex procedural learning as well as visual discrimination. The mice had to first complete the four stages of pretraining (see methods). Pretraining results showed no differences between the genotypes in survival curves when analyzed based on day‐by‐day performance changes (Figure 6A; Mantel‐Cox *X* ^2^(1) = 1.102, *p* = 0.2939). In a direct comparison of the total number of days required to complete pretraining, there was no difference found between the groups either (Figure 6B; *t* = 1.303, *df* = 25, *p* = 0.2044). However, the heterozygotes did demonstrate a decrease in the number of trials needed to progress through pretraining (Figure 6C; *t* = 2.677, *df* = 25, *p* = 0.0129). This suggests that the heterozygotes may have been more efficient with each training session compared with the wild‐type mice, using fewer overall trials to learn each stage.

Once mice satisfied the pretraining stages, they were advanced to the visual discrimination task. In this task, mice were rewarded for interacting with one image (S+) and punished with a timeout period for interacting with a different image (S−). Comparison between the groups revealed no significant difference in performance, as illustrated by a survival curve (Figure 6D; Mantel‐Cox *X* ^2^(1) = 0.5957, *p* = 0.4402). Similarly, there was no difference noted in the total number of days to reach criterion (Figure 6E; *t* = 0.06926, *df* = 15, *p* = 0.9457) or in the number of trials (Figure 6F; *t* = 0.2771, *df* = 15, *p* = 0.7855). These results suggest that the wild‐type and heterozygotes exhibit a similar ability to visually discriminate between two distinct images.

## 4 DISCUSSION

In this study, we demonstrate that reduced expression of *DSCAM*, because of the heterozygosity of a LoF mutation, can produce several behaviors that resemble those observed in children and adults with ASD. First, loss of one copy of *DSCAM* led to a significant increase in activity and anxiety‐like behavior when exploring a novel environment. It also produced difficulties in motor coordination. Additionally, the mutant mice displayed deficits in spatial learning and memory across multiple domains, including short (i.e., working) and long‐term memory. Third, fear learning was acutely affected in *DSCAM* heterozygous mice, mirroring the lack of awareness to danger that is reported in children with ASD. However, decreased *DSCAM* expression did not result in a deficit in procedural learning. The close similarity to behaviors observed in ASD, particularly in children, suggests that *DSCAM* LoF mutations and the mechanisms involved may underlie those phenotypes.

### 4.1 Hyperactivity

Diagnosis of ASD typically occurs in early childhood, often by 3–5 years old.^37^ Multiple screening tests exist, but all require a thorough behavioral evaluation which is often repeated at several key stages of development to confirm the diagnosis.^38^ However, many of the symptoms display considerable overlap with other commonly diagnosed childhood disorders such as attention‐deficit hyperactivity disorder (ADHD), obsessive‐compulsive disorder (OCD), and bipolar disorder (BD).^39^ It is not uncommon for ASD to be co‐diagnosed with one or more of these disorders. In fact, it has been reported that 40%–70% of ASD cases qualify and are co‐diagnosed for ADHD.^40^ One of the primary reasons for the overlap is hyperactivity, which is distinctly present in ADHD but also a prominent symptom of ASD. Therefore, this is a key phenotype to recapitulate in a mouse model of ASD. Here, we found that that *DSCAM* heterozygous mice traveled significantly more distance during exploration of the novel environment (Figure 1A,B). Hyperactivity was also noted in the Y‐maze with increased number of arm entries and distance traveled (Figure 2B,C), and in the Morris water maze with an increased swim speed (Figure 3E). Our results conflict with those reported by Lim et al., where no hyperactivity was noted in open field exploration.^32^ However, this discrepancy may be explained by the mouse model, as Lim et al. used a Nestin‐Cre to specifically target neural stem cells (NSCs), whereas our model represents a global heterozygosity of *DSCAM*. Therefore, our results show that a global loss of one copy of *DSCAM* leads to hyperactivity in exploratory behavior akin to that observed in childhood ASD.

### 4.2 Motor coordination

Individuals with ASD have long been documented as expressing peculiar motor behaviors, specifically restricted repetitive behaviors (RRBs) such as body rocking or hand flapping.^41^ These RRBs are a core feature of the DSM‐V criteria to qualify for ASD diagnosis.^42^ However, the motor phenotype of ASD extends beyond these repetitive movements. There is evidence that broader impairments in both gross and fine motor skills are present in children with ASD.^43^ Furthermore, these impairments may correlate with the degree of intellectual disability,^44^ with more cognitively impaired individuals displaying more significant alterations in motor skills. The results of these studies, among others,^45^, ^46^ strengthen the argument that motor performance should be tested and included in the diagnostic criteria for ASD. Therefore, it is important that motor behavior impairments be expressed and studied in mouse models of ASD. We evaluated our *DSCAM* heterozygous mice in the rotarod behavioral task to probe their locomotor coordination. The mice had substantial difficulty with the rotarod task and were not able to improve upon their performance with additional training (Figure 1E,F). As this task requires active balance and adjustment to an accelerated rotation, any impairment in motor skills or praxis will negatively affect performance. These results align with reported studies that demonstrate motor coordination deficits in children with ASD.

### 4.3 Short‐ and long‐term memory

Despite the common misconception, autism is not often coupled with savant syndrome. Estimates suggest only 1 in 10 individuals with ASD show savant‐like traits.^47^ In fact, intellectual disability (ID), of varying severity, is more frequently observed in ASD and co‐occurring rates may be as high as 50%–70%.^48^ Cognitive impairments vary among individuals, but the majority of ASD cases with ID show deficits in domains such as executive function, language, working memory, and spatial and episodic memory.^49^ This type of impairment profile strongly suggests the prefrontal cortex and hippocampus as brain regions that may be affected in ASD. The first area that we sought to evaluate in the *DSCAM* heterozygous mice was working memory in the Y‐maze (Figure 2), where we observed deficits in correct alternations that placed them significantly below chance (Figure 2D). Furthermore, the heterozygotes had more direct revisit errors (Figure 2E), signaling that these mice were unable to form a proper working memory map of the maze – regardless of the spatial cues available to them. It is worth noting that the *DSCAM* heterozygous mice were hyperactive during the task, with a greater number of arm entries and distance traveled (Figure 2B,C). This may have also contributed to their poor performance, if their hyperactivity had impeded their ability to formulate a cognitive map of the maze. It is therefore important to consider that ADHD, with which ASD is often co‐diagnosed, also carries substantial impairments in working memory and it may be difficult to separate the two disorders in this domain.^50^

While working memory deficits are well‐documented in ASD, deficits in long‐term memory, specifically within the realm of spatial and episodic memory, are less understood. Children and adults with ASD typically show difficulties within these domains, such as an inability to retrieve details of life events and often not including themselves in stories from the past. The source of this impairment had been attributed to the social cognition deficits that are a hallmark of ASD,^51^, ^52^ but new research suggests that there are distinct disruptions in explicit learning and memory that may be independent of those reported in social cognitive processes.^53^, ^54^ If so, focusing on the hippocampus and behaviors specific to disruptions of hippocampal structure and processes may bear fruit for understanding ASD cognitive deficits. In this study, the *DSCAM* heterozygous mice did show a subtle impairment in the Morris water maze task (Figure 3), which is heavily dependent on the hippocampus. While the mutant mice were able to perform the task and significantly outperform chance in the probe trials, they exhibited a less selective search strategy (Figure 3F,G). These results demonstrate that *DSCAM* heterozygotes can still learn and recall the platform location but less effectively so, which is reminiscent of ASD individuals' ability in episodic memory tests. When coupled with Chen et al.'s findings that a complete knockout of *DSCAM* produces social recognition impairments,^33^ these results agree with the theory that social cognitive and spatial learning deficits coexist in ASD and may be independent phenotypes.

### 4.4 Fear learning

Another aspect of learning that appears to be differentially affected in ASD is fear learning, and a common symptom of ASD in children is a “muted” fear response.^55^ This behavior is difficult to interpret as it could be because of multiple factors. There are known impaired domains in ASD that could be playing a role, such as poor social recognition and emotional dysregulation^56^ or a deficit in spatial learning implicating the hippocampus. There are also less studied aspects of ASD that may be contributing factors, such as improper consolidation of fear memories in the anterior cingulate cortex or a more direct disruption of the emotional learning process within the amygdala. All of these components are involved in fear circuitry,^57^ and it seems possible that a combination of affected components is likely. Brain imaging studies have shown that adults with ASD exhibit a heightened anxiety and increased reactivity in the amygdala while in safe contexts, and an inability to properly differentiate between safe and threatening conditions.^58^ Researchers postulate that this may be because of saturation of the amygdala activity through heightened baseline arousal, and therefore fear cannot be appropriately contextualized. Moreover, there appears to be an inverse correlation between ASD symptom severity and the strength of the fear response,^59^ suggesting a direct relationship between the behavioral symptoms and possible disruption of the neurocircuitry in the amygdala‐hippocampus‐cortex fear network. The molecular underpinning of this relationship needs further investigation, but fear learning does indeed appear to be impaired in ASD. In this study, we detected a significant deficit in fear learning and memory within the *DSCAM* heterozygous mice that extended to 30 days post‐exposure (Figure 4B–E). The experimental and control groups displayed similar responses to the cued tone (Figure 4F), signaling that the deficit is likely in context‐specific memory, and therefore likely dependent on the hippocampal processes that are encoding those context‐based memories. Determining the extent to which the amygdala may play a role in this phenotype, and whether loss of one copy of *DSCAM* drives disruptions in the amygdala among other regions in fear neurocircuitry would be of great interest and could inform mechanisms in ASD.

### 4.5 Implicit learning

ASD is a neurodevelopmental disorder that affects the way individuals process the world, both socially and non‐socially, to learn and make decisions. The previously discussed studies outline the varying degrees of cognitive impairment present in ASD, impacting numerous domains. Nonetheless, many autistic individuals succeed in academic settings and beyond, despite these learning difficulties. This may be explained by the fact that implicit memory, such as perceptual and procedural memory, is seen to be unaltered in ASD.^60^, ^61^ Combining these findings, a “see‐saw effect” hypothesis has emerged in the field, suggesting that intact implicit memory systems act to compensate for the impairment seen in explicit systems such as episodic memory.^60^ With ASD rising in the general population^1^ there is an increased demand to understand the disorder and develop strategies and methods to accommodate ASD children in school^62^ and adults in the workplace.^63^ An example of which may be a greater focus on active learning and allowing autistic individuals to learn at their own pace. Indeed, active learning strategies have been seen to improve episodic memory in children with ASD.^64^ This may help explain our findings in the touchscreen systems (Figure 6), where the *DSCAM* heterozygous mice performed as well as, or even slightly better than, the control mice. With procedural learning intact, the pretraining stage was not an impediment and the *DSCAM* heterozygotes even completed this stage in less overall trials. While not every mouse completed the visual discrimination portion of the experiment, there was no difference between each group's performance in those that did complete the experiment (Figure 6D–F). The nature of this task, where each daily session allows the mouse to be undisturbed for an hour and learn at their own pace, may have provided the right conditions for optimal performance despite previously illustrated cognitive deficits, as has been argued for human cases of ASD. Alternatively, it is possible that the trend suggesting that the *DSCAM* heterozygous mice outperformed their wild‐type littermates reflects some sort of perseverative tendencies in the heterozygotes.

### 4.6 Sex differences and future directions

Sexual dimorphism in neurodevelopmental disorders is well‐documented^65^ with some disorders displaying sex differences in the onset of symptoms, symptom presentation, and overall prevalence. In fact, many disorders are seen to exhibit a male bias, including ADHD, intellectual disability, and communication disorders.^66^ ASD is no exception, with the estimated prevalence in children under 8 years old supporting a male/female ratio of 3.8:1.^1^ Numerous hypotheses to explain this bias have been proposed, with most positing sex‐specific risk genes or pathways that may be differentially influenced or disrupted, but results are often difficult to replicate.^67^ There is also an emerging theory that ASD in females may go underdiagnosed because of differences in symptom presentation and comorbidities that mask the correct diagnosis,^68^ suggesting that the male bias in ASD may be overestimated. Although the present study was determined to be underpowered to assess sex differences, future research may clarify whether the *DSCAM* ^2J^+/− exhibit a bias toward males that aligns with the reported bias present in human cases of ASD.

## 5 CONCLUSIONS

Growing evidence shows that *DSCAM* acts in a dose‐dependent manner. While the overexpression of *DSCAM* may be contributing to cognitive impairments observed in Down syndrome, its underexpression could be a pathway to autistic‐like phenotypes. Our results established that *DSCAM* heterozygous mice exhibit many behaviors that are reminiscent of those observed in ASD, such as hyperactivity, motor coordination abnormalities, and learning and memory deficits. These findings, coupled with previous research, provide compelling evidence that *DSCAM* LoF mutations may be an avenue through which ASD‐related behaviors are produced and advocate for *DSCAM* ^2J^+/− as a model for ASD research and therapeutic testing.

## AUTHOR CONTRIBUTIONS

**Ryan C. Neff**: Investigation; formal analysis; writing‐original draft; writing‐review & editing; validation; visualization. **Katherine A. Stangis**: Investigation; formal analysis; resources; writing‐review & editing. **Ujjawal Beniwal**: Investigation; formal analysis. **Ty Hergenreder**: Resources; writing‐review & editing. **Bing Ye**: Conceptualization; writing‐review & editing; funding acquisition. **Geoffrey G. Murphy**: Conceptualization; validation; writing‐review & editing; funding acquisition; supervision.

## FUNDING INFORMATION

This work was supported by grants from NIH (R01AG052934 to Geoffrey G. Murphy, and R01EB028159 and R21NS094091 to Bing Ye), a seed grant from Brain Research Foundation to Bing Ye, and Protein Folding Disease Initiative of the University of Michigan to Geoffrey G. Murphy and Bing Ye, and the University of Michigan Rackham Research Grant to Ryan C. Neff.

## CONFLICT OF INTEREST STATEMENT

The authors declare no conflicts of interest.

## ETHICS STATEMENT

The animal study and protocols were reviewed and approved by the University of Michigan Institutional Animal Care and Use Committee (IACUC) and in accordance with the National Research Council Guide for the Care and Use of Laboratory Animals (NIH).

## Supporting information


**Figure S1:** *DSCAM* ^2J^+/− heterozygotes do not differ in thermal nociception. Pain sensitivity was evaluated using the hot plate test. (A) The latency to hindlimb lick indicated there were no significant differences between the groups in their thermal nociception, or tolerance to heat pain. Results are presented as Mean ± SEM. Sample size *DSCAM* ^2J^+/− *n* = 7 and WT *n* = 9. Comparison between heterozygotes and WT mice was analyzed by a two‐tailed *t*‐test.

## Data Availability

The data that support the findings of this study are available from the corresponding author upon reasonable request.
