# Supplementary material for: Cognitive behavioral phenotyping of DSCAM heterozygosity as a model for autism spectrum disorder
Source: Genes Brain Behav. 2024 Sep 18;23(5):e70002. doi: 10.1111/gbb.70002 (PMC11410459; doi:10.1111/gbb.70002)
Supplement: Supplementary file 1 — Figure S1: DSCAM 2J+/− heterozygotes do not differ in thermal nociception. Pain sensitivity was evaluated using the hot plate test. (A) The latency to hindlimb lick indicated there were no significant differences between the groups in their thermal nociception, or tolerance to heat pain. Results are presented as Mean ± SEM. Sample size DSCAM 2J+/− n = 7 and WT n = 9. Comparison between heterozygotes and WT mice was analyzed by a two‐tailed t‐test. [file GBB-23-e70002-s001.docx]

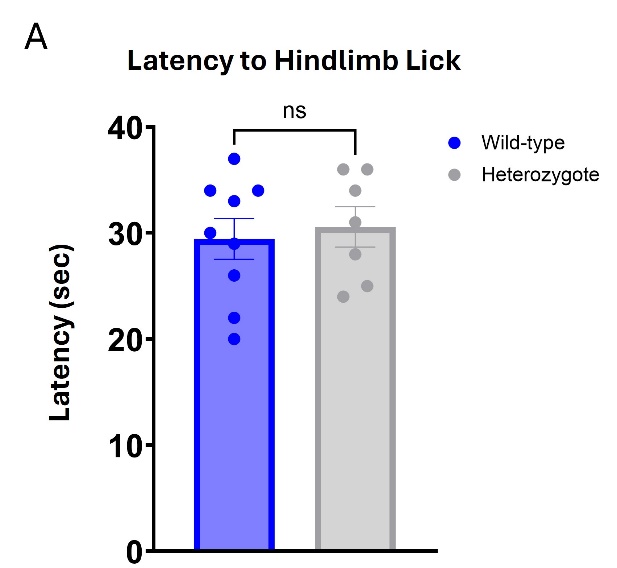


**Supplementary Figure 1**

***DSCAM*^2J^+/- heterozygotes do not differ in thermal nociception.** Pain sensitivity was evaluated using the hot plate test. (**A**) The latency to hindlimb lick indicated there were no significant differences between the groups in their thermal nociception, or tolerance to heat pain. Results are presented as Mean ± SEM. Sample size *DSCAM*^2J^+/- n = 7 and WT n = 9. Comparison between heterozygotes and WT mice was analyzed by a two-tailed *t*-test.
